# Complex Cardiovascular Management in an 80-Year-Old Female With Gastrointestinal Bleeds and Klatskin Tumor: A Case of ST-Elevation Myocardial Infarction (MI) Management Complicated by Severe Anemia and Post-MI Ventricular Septal Defect Development

**DOI:** 10.7759/cureus.75699

**Published:** 2024-12-14

**Authors:** Jesse O'Rorke, Greyson Butler, Ramesh Chandra

**Affiliations:** 1 Osteopathic Medicine, Lee Health, Fort Myers, USA; 2 Osteopathic Medicine, Lake Erie College of Osteopathic Medicine, Bradenton, USA; 3 Interventional Cardiology, Lee Health, Fort Myers, USA

**Keywords:** acute chest syndrome (acs), acute gi bleed, dual-antiplatelet therapy (dapt), emergency echocardiography, klatskin tumor, post mi complication, post mi vsd, st-segment elevation myocardial infarction (stemi), vsd: ventricular septal defect

## Abstract

Managing acute coronary syndrome (ACS) in patients with a recent history of gastrointestinal bleeding presents a unique and challenging clinical dilemma, necessitating a careful balance between minimizing ischemic risk and avoiding potentially life-threatening rebleeding. Standard treatment for ACS typically involves dual antiplatelet therapy (DAPT) to prevent recurrent thrombotic events. However, in patients with recent gastrointestinal hemorrhage or significant anemia, these therapies may substantially increase the risk of life-threatening bleeding, complicating the decision-making process and often leading to conservative management strategies.

In this case, we describe the presentation and management of an 80-year-old female with a history of Klatskin tumor resection, duodenal ulcer, and recurrent gastrointestinal bleeding who was admitted with symptoms suggestive of ACS. An electrocardiogram (EKG) revealed ST elevation in anterolateral leads, raising concerns for an acute myocardial infarction (MI). However, given her critically low hemoglobin (5.7 g/dL) and recent history of gastrointestinal hemorrhage, the decision was made to forgo aggressive interventions such as percutaneous coronary intervention (PCI) and DAPT. A conservative approach, including aspirin monotherapy and supportive care, was prioritized due to the high risk of rebleeding. Despite initial stabilization with transfusions, the patient later developed hemodynamic instability and was found to have a large ventricular septal defect (VSD) on echocardiography, ultimately leading to her demise.

This case underscores the complexities of managing ACS in patients with severe anemia and recent gastrointestinal bleeding, where standard ACS protocols may be contraindicated. It highlights the importance of individualized, multidisciplinary treatment strategies and shared decision-making with patients and families to optimize care while aligning with the patient's overall health goals. In high-risk cases like this, a conservative approach may be warranted, even when it conflicts with traditional aggressive treatment pathways. The lessons learned from this case reinforce the need for flexibility and critical thinking in navigating the delicate balance between preventing ischemic complications and avoiding catastrophic bleeding in vulnerable patients.

## Introduction

Managing acute coronary syndrome (ACS) in patients with a recent history of gastrointestinal bleeding presents a complex clinical challenge, as these cases require a delicate balance between reducing ischemic risk and minimizing the potential for life-threatening rebleeding. Typically, patients with ACS benefit from dual antiplatelet therapy (DAPT) and anticoagulation, which are used to prevent recurrent thrombotic events. However, these therapies also substantially increase bleeding risk, particularly in patients who have recently experienced a gastrointestinal hemorrhage or have anemia. For patients with low hemoglobin or other indications of anemia, the bleeding risk can become prohibitive, complicating the standard approach and necessitating individualized care. In many cases, this leads to the adoption of a conservative strategy, prioritizing symptom management over aggressive intervention when the likelihood of bleeding outweighs the anticipated benefit from procedures such as percutaneous coronary intervention (PCI). Emerging evidence also suggests that proton pump inhibitors (PPIs) may be beneficial in reducing upper gastrointestinal bleeding in patients requiring DAPT, although their role remains an adjunctive measure rather than a definitive solution [1]. These challenging cases call for a collaborative, multidisciplinary approach to align treatment with the patient's health status and goals of care.

Further complicating the management of ACS in these high-risk patients is the potential for structural cardiac complications following myocardial infarction (MI), such as ventricular septal defect (VSD). VSD is a rare but severe complication that arises when infarcted myocardial tissue ruptures, forming an abnormal connection between the left and right ventricles. This defect can lead to hemodynamic instability and heart failure, particularly in patients already compromised by recent bleeding and limited options for aggressive cardiac intervention. In patients with hemodynamically significant VSDs, temporary mechanical circulatory support devices may be considered to stabilize the patient until definitive treatment can be undertaken, although their use is highly individualized [2]. When VSD occurs in the setting of a recent gastrointestinal bleed, the treatment dilemma intensifies, as even conservative measures may be inadequate to stabilize the patient, and surgical options may be contraindicated. These complex cases underscore the importance of a careful, case-by-case assessment of risks and benefits, and they highlight the need for clinicians to adopt a flexible and patient-centered approach.

## Case presentation

An 80-year-old female with a past medical history of Klatskin tumor status post resection and radiation therapy, possible cirrhosis of the liver status post partial liver resection, duodenal ulcer, gastrointestinal bleeding, and anemia presents to the emergency department with a chief complaint of nausea and fatigue. The patient’s history was significant for a recent hospitalization for a gastrointestinal bleed with black tarry stools. She underwent esophagogastroduodenoscopy (EGD) with a finding of a large fibrin clot at the duodenal bulb with no definite changes of malignancy noted on histopathology. At that time, she was started on oral proton pump inhibitor therapy. During the previous hospitalization, her hemoglobin was between 4-5 g/dL, and she received a packed red blood cell transfusion, getting her hemoglobin up to 7.6 g/dL at the time of discharge. 

**Figure 1:**
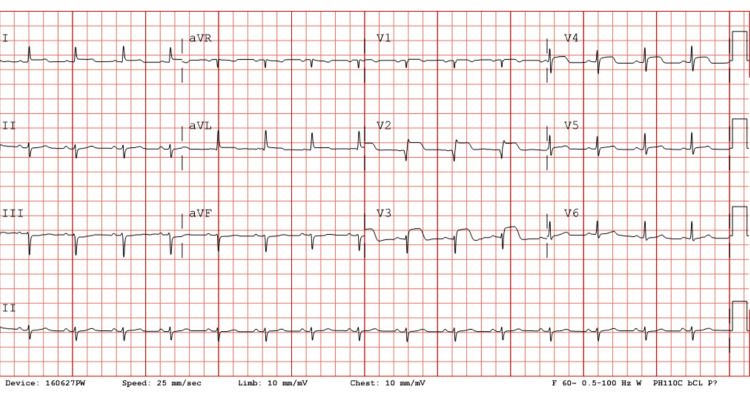
Patient’s EKG showing significant ST elevation in leads V2-V5 and reciprocal ST segment depression in the inferior leads

The patient described her fatigue as worsening over the past week, with a black tarry stool occurring two days before the presentation. She reports she has not had a bowel movement since. The patient was noted to be pale on arrival, with continued reporting of fatigue and nausea; she denied chest pain at that time. An EKG was performed on arrival, which was significant for ST-segment elevation in the anterolateral leads with reciprocal ST-segment depression in the inferior leads, as seen in Figure 1. The patient’s laboratory work was also significant for a high-sensitivity cardiac troponin I of 1,483. At this time, the interventional cardiology team was consulted for emergent cardiac catheterization. When the patient was examined by the cardiology team, she remained chest pain-free. The patient became transiently hypotensive after receiving nitroglycerin, but her blood pressure was responsive to an IV fluid bolus.

Considering the patient’s recent gastrointestinal bleeding, after extensive discussion, aspirin 325 mg was administered. It was decided not to proceed with heparin administration because of a recent gastrointestinal bleed and dropping hemoglobin. Hemoglobin was measured in the emergency room to be 5.7 g/dL, which is lower than her previous discharge hemoglobin. Hence, when analyzing the full clinical picture, the patient was determined to be a poor candidate for cardiac catheterization and PCI. It was discussed that if the patient were to receive DAPT, which is defined as aspirin 81 mg and a P2Y12 inhibitor, and a large dose of anticoagulation during the procedure, she would be at a potentially life-threatening risk of gastrointestinal bleeding. In other words, the risk of periprocedural complication/bleeding outweighed the reward that could have been gained from performing PCI. Additionally, the cardiology team did not recommend therapeutic anticoagulation or DAPT; the recommendation was to continue 81 mg of aspirin daily if tolerated. Comfort measures and symptomatic treatment were recommended by the cardiology team.

Shortly after the cardiology team made their decision and discussed it with the patient and her family, the patient and her family requested a Do Not Resuscitate order. The patient received a transfusion of two units of packed red blood cells; her hemoglobin improved to 8.5 g/dL post-transfusion. The gastroenterology team commented that there are no signs of active gastrointestinal bleeding; their recommendation was symptomatic treatment and medical optimization with resuscitation and transfusions with a hemoglobin goal of at least 8 g/dL. They opted for no further invasive procedures at that time, along with administration of intravenous proton pump inhibitor therapy and continued Carafate.

**Figure 2:**
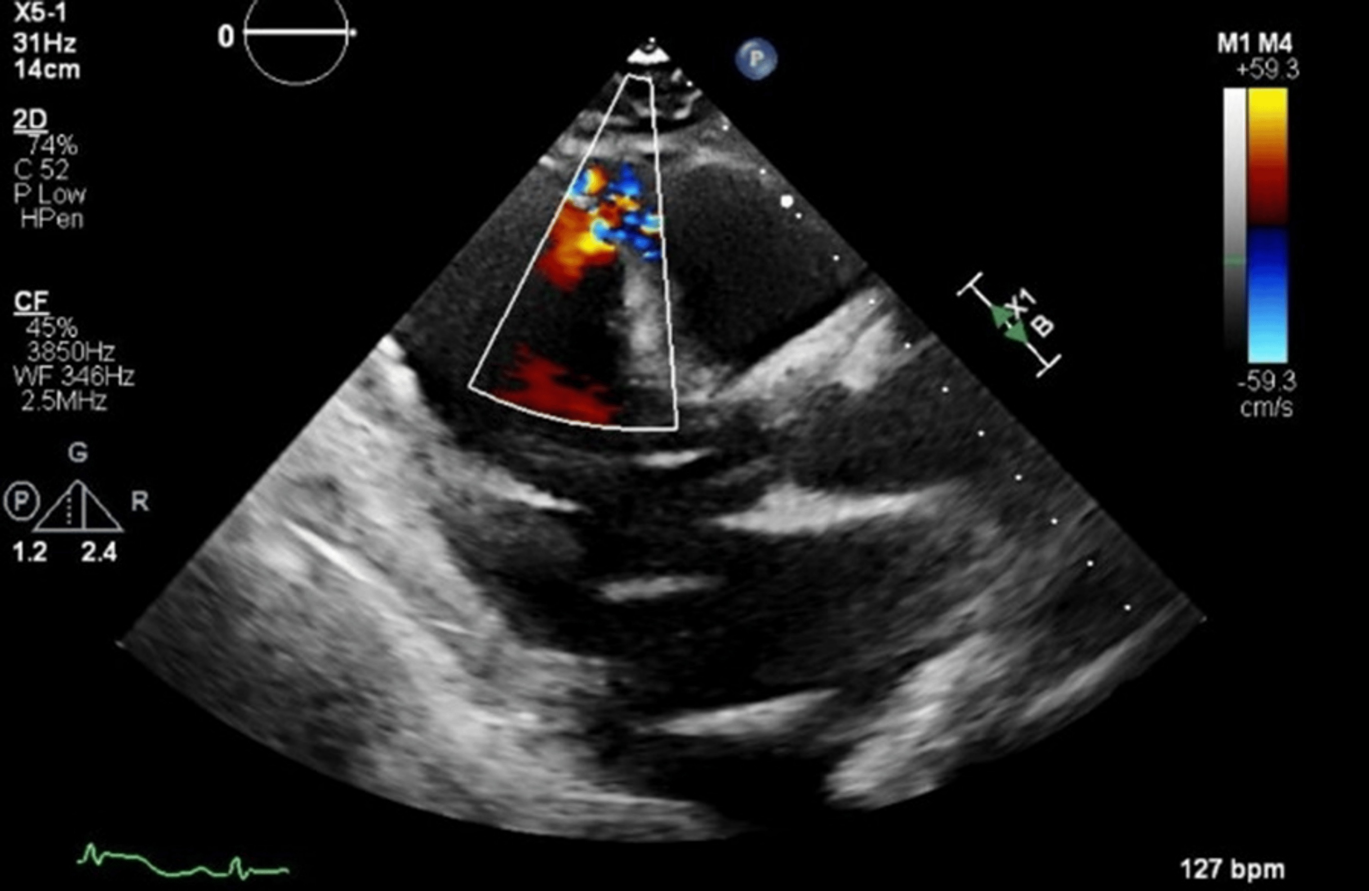
Still image from the patient’s echocardiogram with a large ventricular septal defect (VSD) with left to right shunting towards the apex

The next morning, the patient’s blood pressure trended down, and telemetry showed the patient became bradycardic, which proceeded to asystole. At this time, the patient was found unresponsive, and the emergency response team was called at 0854; it was noted that the patient changed the code status to “full code” overnight. The cardiology team responded at the bedside and spoke to her daughter, who changed the patient’s code status to “allow natural death” (AND) after about five minutes of cardiopulmonary resuscitation and endotracheal intubation. At the daughter’s request, resuscitation efforts ended at that time. An echocardiogram was performed at the bedside, showing a large VSD with left-to-right shunting toward the apex, suggesting myocardial injury that likely began around one week ago, as shown in Figure 2. The patient was pronounced dead at 0903.

An important note from the patient’s history is that she had a resection of her Klatskin tumor one year prior to presentation, as mentioned above. This resection was complicated by the development of a portal vein thrombosis for which Eliquis therapy was initiated. It was subsequently discontinued due to a gastrointestinal bleed.

## Discussion

In patients with low hemoglobin levels, especially those with recent gastrointestinal bleeding, the risk of further bleeding increases significantly, presenting a complex challenge in concurrently managing ischemic events and anemia. Low hemoglobin not only indicates reduced oxygen-carrying capacity but also suggests an impaired hemostatic balance, as diminished red blood cell mass can hinder effective clot formation, potentially worsening bleeding tendencies [3]. Severe anemia, particularly with hemoglobin levels below 7 g/dL, is associated with higher mortality and adverse outcomes in patients with gastrointestinal hemorrhage and coexisting cardiovascular disease [4]. Consequently, decisions regarding interventions - such as DAPT or anticoagulation - must carefully balance the increased bleeding risk against the benefits of managing ischemic events. Clinical strategies typically aim to stabilize hemoglobin to safer levels, between 7 and 9 g/dL, to reduce bleeding risk while cautiously/medically addressing ischemic symptoms [5]. Transfusion strategies that balance these risks can help stabilize hemoglobin without exposing patients to complications related to high-volume transfusions, such as transfusion-associated overload or immune reactions [6]. This delicate balance highlights the need for individualized, conservative management, especially in high-risk patients, such as elderly individuals with multiple comorbidities.

Anemia can lead to various EKG changes, resulting from myocardial ischemia and infarction due to the reduced oxygen-carrying capacity and increased myocardial oxygen demand associated with low hemoglobin levels. These EKG alterations can include ST segment depression, T-wave inversions, and other nonspecific changes that appear like myocardial ischemia, even in the absence of obstructive coronary artery disease [7]. Distinguishing anemia-related EKG changes from true ischemic events is crucial, as misinterpretation could lead to unnecessary invasive procedures or therapies that carry additional risks, especially in patients with a history of bleeding or other comorbidities. A careful assessment of EKG findings, alongside clinical evaluation and laboratory data, can help clinicians avoid potentially harmful interventions in cases of demand ischemia and adopt a more conservative management approach when appropriate. Understanding anemia's potential to produce changes on EKG emphasizes the importance of a thorough, individualized approach to cardiac evaluation in anemic patients with complex medical profiles.

A VSD is a rare but serious complication of acute MI, typically occurring within four to seven days after an ischemic event due to a rupture of infarcted myocardial tissue. This complication is most common in patients with large transmural infarctions, particularly those involving the left anterior descending artery, where elevated pressure and blood flow create shear stress that can result in septal rupture [8]. Specifically, the typical process leading to septal rupture involves coagulative necrosis of ischemic tissue accompanied by neutrophilic infiltration, which progressively thins and weakens the septal myocardium [9]. A post-MI VSD creates a left-to-right shunt, leading to acute heart failure and severe hemodynamic instability, with a high risk of mortality if untreated [10]. Clinically, patients often present with signs of cardiogenic shock, a new harsh blowing holosystolic murmur, and imaging findings indicating shunt flow across the septum. Surgical repair is generally required, although mortality remains high despite intervention, emphasizing the importance of early recognition and management [11]. The risk of VSD following MI has declined with advancements in early revascularization, yet it remains a concern, particularly in patients with delayed presentation or those ineligible for immediate intervention [12]. A thorough understanding of the pathophysiology and presentation of this complication is essential for prompt diagnosis and potentially life-saving treatment.

Klatskin tumors, or perihilar cholangiocarcinomas, are rare malignancies located at the junction of the right and left hepatic ducts, which can lead to biliary obstruction and associated complications. The tumor's anatomic position can impede biliary drainage, leading to cholestasis, jaundice, and eventually cholangitis if untreated [13]. Additionally, these tumors frequently infiltrate nearby vascular structures, including the portal vein and hepatic artery, further complicating surgical resection and increasing the risk of portal hypertension [13,14]. Treatment is challenging because Klatskin tumors are often diagnosed at an advanced stage due to their asymptomatic progression; thus, curative resection or liver transplantation may not be feasible for many patients [15]. Palliative treatments such as biliary drainage, stenting, and selective use of radiation or chemotherapy are often employed to manage symptoms and delay progression [15,16]. However, these approaches can lead to additional complications, including infection, bleeding, or hepatic insufficiency [16]. The complexity of managing Klatskin tumors underscores the need for a multidisciplinary approach, aiming to balance symptom control with minimizing treatment-associated risks, and highlights the poor prognosis associated with these tumors.

## Conclusions

The management of ACS in patients with recent gastrointestinal bleeding presents a significant therapeutic dilemma. When severe anemia is part of the equation, it greatly complicates matters, as the risk of severe bleeding from giving loading doses of antiplatelet therapy, as is required when performing PCI, usually outweighs the benefit to be gained from regaining flow. As seen in this case, a conservative approach was taken in lieu of the normal aggressive strategy of attempting PCI in a patient displaying signs of ST elevated MI on their EKG. The patient then went on to develop a large VSD, most likely secondary to the MI, which was plausibly the factor that precipitated her demise.

This case underscores the critical importance of individualized risk stratification, multidisciplinary collaboration, and clear communication with patients and families to align treatment strategies with the overall goals of care. By balancing ischemic and bleeding risks, clinicians can navigate complex cases to optimize outcomes even in the most challenging clinical contexts. This article should serve as a reinforcement of the difficult decisions clinicians make on a daily basis to optimize care and comfort and that even when the right decision is made at the time, poor outcomes can still follow.
